# Adding a Piece to the Puzzle? The Allocation of Figurative Language Comprehension into the CHC Model of Cognitive Abilities

**DOI:** 10.3390/jintelligence12030029

**Published:** 2024-03-01

**Authors:** Andra Biesok, Matthias Ziegler, Christiane Montag, Ivan Nenchev

**Affiliations:** 1 Psychology Department, Faculty of Life Science, Humboldt-Universität zu Berlin, 10117 Berlin, Germany; matthias.ziegler@hu-berlin.de; 2 Department of Psychiatry and Psychotherapy, Campus Charité Mitte—Universitätsmedizin Berlin, Corporate Member of Freie Universität Berlin, Humboldt-Universität zu Berlin, and Berlin Institute of Health, 10117 Berlin, Germany; christiane.montag@charite.de (C.M.); ivan.nenchev@charite.de (I.N.)

**Keywords:** figurative language comprehension, CHC model, personality, openness

## Abstract

The study aimed to investigate the allocation of figurative language comprehension (FLC) within the Cattell–Horn–Carroll (CHC) model of cognitive abilities, using three newly developed tests: the Reverse Paraphrase Test (RPT), the Literal Paraphrase Test (LPT), and the Proverb Test (PT). The analysis of a sample of 909 participants revealed that the RPT and LPT measured a unidimensional construct of FLC, while the PT was excluded due to insufficient fit. Combining RPT and LPT items, various models were evaluated, with a bifactor S-1 model showing the best fit, indicating the influence of a general factor (representing FLC) and test-specific method factors. The study explored FLC allocation within the CHC model, supporting its consideration as a distinct factor under the g factor. Examining the nomological network, significant correlations emerged between the Intellectual Curiosity and Aesthetic Sensitivity facets of Openness and FLC, which were comparable in size to the relation with general ability. In conclusion, the study enhances the understanding of FLC within the CHC model, advocating its recognition as a distinct factor. Correlations with Openness facets suggest valuable insights into the interplay between cognitive abilities and personality, necessitating further research for a deeper exploration of this relation.

## 1. Introduction

Much of human thinking is conceptualized through metaphors, proverbs, irony, and other instances of figurative language (Lakoff and Johnson 2003), which means that people are exposed to figurative language early in their development and have predominantly fewer problems understanding and producing it later in life. Figurative language comprehension influences social relationships, social participation, and educational achievement (Cain et al. 2005; Kerbel and Grunwell 1997; Swineford et al. 2014). As a result, the inability to efficiently understand this form of communication may contribute to the social exclusion of these individuals (Mitchell and Crow 2005) and may seriously affect an individual’s life. Even though psychological research has produced a plethora of knowledge on figurative language comprehension (Gibbs 1994; Glucksberg and McGlone 2001; Kintsch 2000), and its creation and production (Chiappe and Chiappe 2007; Pereira de Barros et al. 2010; Pierce and Chiappe 2009; Silvia and Beaty 2012) in healthy and especially clinical samples, there is still an ongoing discussion about the construct in general as well as its location in a broader nomological network of abilities. Moreover, personality traits might also be relevant within the nomological network, in particular the personality trait Openness as it is linked to creativity which has been shown to be beneficial to metaphor creation (Beaty and Silvia 2013; Silvia and Beaty 2012; Sutin 2015). Creative people tend to prefer the use of complex and unconventional metaphors when speaking figurately, and figurative language may perhaps be the most common expression of creativity in everyday life (Carter 2016). Whether this personality trait also relates to figurative language comprehension will be explored here. Thus, the current paper addresses these gaps in research by first testing the internal structure of different tests operationalizing figurative language comprehension and, second, by testing the position of those tests within parts of the Cattell–Horn–Carroll (CHC) model of cognitive abilities (Schneider and McGrew 2018). And, finally, the paper explores the relation between personality traits and figurative language to add personality into the puzzle of figurative language comprehension. 

## 2. Different Definitions and Models of Figurative Language Comprehension and Problems Resulting from This

Definitions of figurative language are heterogeneous (Glucksberg and McGlone 2001), which is partly due to the different disciplines of the researchers (for example: linguistics, psychology, and philosophy) who have studied figurative language (Gibbs and Colston 2007, 2012; Ortony 1993; Roberts and Kreuz 1994; Simpson 1993). In general, figurative language includes the following: metaphors, proverbs, idioms, irony, and sarcasm, which exist in both spoken and written language (Burgers et al. 2012; Corts and Meyers 2002; Gibbs 1994, 2000; McCarthy and Carter 2004), and one common aspect that all definitions of figurative language share is the description as “speech where speakers mean something other than what they literally say” (Gibbs and Colston 2012, p. 1). 

Furthermore, the individual categories grouped under the term ‘figurative language’ have heterogeneous definitions as well. According to Lakoff and Johnson (2003), metaphors are statements that often relate a more abstract concept to a more concrete one. Metaphors are classified as a higher-order concepts encompassing other structures such as similes and analogies (Barnden 2010). They serve as a descriptive mechanism of communication, wherein they elucidate a specific aspect of a given subject by drawing a conceptual parallel with a related exemplar. These exemplars, often referred to as vehicles, encapsulate abstract relations to a reference concept, which represents the topic at hand.

Understanding the nature of figurative language helps to inform the question as to the psychological processes relevant for figurative language comprehension. For example, comprehending figurative language requires mapping a concept from one semantic source domain (e.g., “jail”) onto another semantic target domain (e.g., “Job” in “My job is a jail”). A variable amount of literal processing is added to the figurative processing because metaphors can but do not have to be literally false. Unlike metaphors, proverbs and idioms can have a literally plausible meaning as well as a (usually more common) figurative one (Gibbs 1994). In particular, idioms can be decomposable so that in some cases, the figurative meaning can at least partly be derived from the literal meaning (as in “to put one’s cards on the table”). Other idioms are opaque, and their meaning is based on convention (as in “to kick the bucket”). Conventional idioms as well as conventional metaphors are more likely to be processed through memory retrieval than to be processed in the moment during reading (Bohrn et al. 2012). Irony is considered a special type of figurative language. An ironic statement could be literally correct, if taken out of context, but the intended meaning of the speaker is different from what is stated literally (Gibbs 1994). To understand an ironic statement, a person must make inferences about the intentions of the speaker and the social context the statement is made in. A subtype of irony is sarcasm or satire, which often has a more negative connotation and is usually used to criticize someone. Feelings and attitudes can be communicated indirectly through an ironic or sarcastic remark; therefore, a person requires skills of perspective taking and integration of social cues to understand irony (Shamay-Tsoory et al. 2005). Thus, a wide array of psychological processes can be assumed to be at play. 

There are different models developed which are specifically designed to account for figurative language comprehension (Filik et al. 2014; Gibbs and Colston 2012; Giora 2003). One of them is a theory by Glucksberg (2003) regarding figurative language comprehension: the property attribution model. This model provides a useful framework for conceptualizing metaphor comprehension but can also be used to understand proverb comprehension, as proverbs are similar to metaphors. The model states that to understand a metaphor, a person has to make an abstract link between a topic and a vehicle by relating similar characteristics (Glucksberg 2003). For example, for the metaphor “My job is a jail”, the person has to create a link between the topic (in this case the job) and the vehicle (in this case the jail). To create that link, the person must have shared conceptual knowledge between the topic and vehicle (Glucksberg et al. 1997). According to that model, people will search their semantic memory for an appropriate vehicle, while an attributive category maintains some characteristics of the topic that can be used to relate to the vehicle. 

The same is true in proverbs, where the person hearing a proverb must decode the proverb by relating similar characteristics and relations as described in the proverb (“The apple does not fall far from the tree”) to a situation that is presented to that person (someone acts in a similar way to their parents).

Furthermore, a more general description of figurative language comprehension describes that there is agreement on two important criteria that should constitute the distinction between figurative and non-figurative language. First, literal statements should express truths (e.g., “Tim is in Canada”), whereas figurative language usually expresses a falsehood (e.g., “Tim is on cloud number nine”), although this distinction is controversial (Gibbs and Beitel 1995). Second, literal language confirms linguistic constraints, whereas figurative language tends to violate them. For example, in the sentence “The car is very thirsty”, the use of the adjective “thirsty” is violated because only living beings can be thirsty.

To summarize, the definitions and models lead to a prevalence of studies which postulate that the interpretation of figurative language requires the recognition, comprehension, and rejection of a figurative utterance’s literal meaning in order to arrive at a secondary, figurative interpretation (Grice 1975; Sperber and Wilson 2002), postulating that the comprehension of figurative language seems to contain processes similar to those defining other language processing abilities while also comprising cognitive processes at the core of understanding figurative language, which are rather unique. Specifically, these ideas strongly suggest that what makes figurative language comprehension unique is the ability to abstract an expression, hold it in mind and/or locate higher-order attributive categories, and ‘fit’ them to the sentence. This requires the ability to ‘distance oneself’ from a sentence, to reformulate and transform it. It involves moving away from a purely content-based consideration without completely disregarding grammatical rules. At the same time, figurative language comprehension requires knowledge and reasoning—both of which are major components in modern models of intelligence structure.

## 3. Modern Conceptualization of Cognitive Abilities

The modern CHC theory (see Carroll 1993, 2003; Schneider and McGrew 2018) is a psychological taxonomy about the structure of cognitive abilities. It integrates multiple theories of intelligence structure to explain interindividual differences in cognitive abilities (Schneider and McGrew 2018). The CHC model links Horn and Cattell’s (1966) theory of fluid and crystallized intelligence with Carroll’s (1993) three-stratum theory (McGrew 2009) and conceptualizes intelligence as a hierarchical structure of abilities, from narrow (specific abilities, such as inductive reasoning) to broad (global abilities, such as fluid intelligence) to general (g). 

The CHC model of cognitive abilities is a robust and dynamic framework that undergoes regular updates to incorporate new or revised ability factors. While the CHC model has expanded to include various other broad domains of intelligence, fluid intelligence (gf) and crystallized intelligence (gc) remain prominently featured due to extensive empirical support for their predictive value in important life outcomes (Alexander and Judy 1988; Deary et al. 2012; Ghisletta et al. 2006; Kuncel et al. 2010; Rolfhus and Ackerman 1999).

Within the CHC model, the combination of comprehension-knowledge (gc), reading and writing (grw), quantitative knowledge (gq), and domain-specific knowledge (gkn) falls under the comprehensive term of acquired knowledge (Schneider and McGrew 2018). Acquired knowledge thus describes the knowledge that a person accumulates over a lifetime, including language, general knowledge, and cultural background information. Fluid intelligence refers to a person’s ability to solve problems, recognize patterns, and accomplish new tasks that do not require prior experience. The recent CHC theory encompasses 17 broad abilities (Schneider and McGrew 2018). These include the domain-general reasoning capacity fluid reasoning (gf) and several acquired-knowledge capacities, including comprehension-knowledge (gc), domain-specific knowledge (gkn), reading and writing (gw), and quantitative knowledge (gq). Furthermore, there are domain-specific sensory abilities corresponding to each of the major senses: visual (gv), auditory (ga), olfactory (go), tactile (gh), and kinesthetic (gk) as well as the psychomotor ability factor (gp). There are three factors related to memory: working memory capacity (gwm), learning efficiency (gl), and retrieval fluency (gr). And finally, there are several abilities related to speed: reaction/decision time (gt), processing speed (gs), and psychomotor speed (gps). The broad and narrow abilities represent separate abilities but function together as an interconnected problem-solving system (Schneider and McGrew 2013). So far, figurative language comprehension is not specifically mentioned in this model. In order to allocate figurative language comprehension (FLC) into the CHC model, it is informative to understand the processes involved. 

## 4. Processes Proposed to Be Involved in the Comprehension of Figurative Language

As outlined above, regarding figurative language comprehension, researchers have mainly focused on uncovering the linguistic and/or cognitive process involved. The linguistic characteristics of figurative language seem to influence how figurative language is comprehended. For example, if the figurative expression is familiar to a person, this results in similar processing speeds for figurative and literal utterances with approximately the same meaning, suggesting that if the figurative meaning has been learned earlier, the figurative meaning is immediately available for more familiar examples of metaphors and ironies. Furthermore, the linguistic context the figurative language is presented in (such as negation, positive or negative quantifiers, and previous uses of figurative language in the same text) seems to have an influence on the speed of figurative language comprehension (Filik and Moxey 2010; Giora et al. 1998, 2007). In addition, the language background and proficiency of a person seem to be an additional individual difference that affects the ability to produce and comprehend figurative language. For example, figurative language seems to be comprehended differently in one’s first compared to one’s second language and between bilinguals and monolinguals (Heredia and Cieślicka 2015; Johnson and Rosano 1993; Titone et al. 2015). 

In addition to the study of linguistic processes, research has also focused on various individual differences, such as cognitive ability and age, concerning the comprehension of figurative language. Several studies have discovered connections between different measures of cognitive abilities, like problem-solving ability and broad memory retrieval, and the capacity to comprehend and generate creative and original metaphors (Gibbs and Colston 2012). The ability to produce metaphors and comprehend metaphorical and sarcastic language has been associated with working memory capacity (gwm), which can be defined as ‘the ability to maintain and manipulate information in active attention’ (Schneider and McGrew 2018, p. 97). Furthermore, exposure to sarcasm has been linked to abstract thinking (Huang et al. 2015), while a preference for engaging in challenging cognitive tasks has been connected to the ability to comprehend metaphors (Olkoniemi et al. 2016). Lastly, age and general background knowledge are significant predictors of comprehension of satire (Boukes et al. 2015; Pfaff et al. 1997; Simpson 2003). 

On the one hand, the broad number of associations between various individual difference variables and the ability to comprehend different types of figurative language, as reported in these studies, underscores the importance of considering multiple factors associated with the individual when examining figurative language comprehension. On the other hand, focusing on testing different models and uncovering diverse processes to be involved in the comprehension of figurative language seem to have clouded the obvious conclusion: All these processes are cognitive ones (even the ones declared as purely ‘linguistic’), suggesting that the comprehension of figurative language might best be positioned in a nomological network consisting of cognitive abilities. This assertion is supported by several researchers themselves, who argue that focusing solely on testing models of figurative language processing is misguided. They contend that figurative language utilizes the same fundamental mechanisms of language processing and comprehension as non-figurative language (Gibbs 1994; Gibbs and Colston 2012; Glucksberg 2003). According to this perspective, any perceived differences in processing figurative and non-figurative language stem from cognitive or non-cognitive differences rather than from a qualitative distinction in how hearers process and interpret non-figurative and figurative meanings.

Consequently, researchers would greatly benefit from an integrated framework that considers and integrates cognitive processes that are crucial for language comprehension overall, including the comprehension of figurative language as well as other cognitive processes described above. This approach acknowledges the regularities and instabilities found in research findings regarding figurative language as reflections of the dynamic nature in which a wide array of variables influence its comprehension and usage (Drnyei and Skehan 2003; Li et al. 2015). Considering that figurative language also entails a creative side, such a framework might also have to incorporate non-cognitive traits, such as the Big Five personality traits.

## 5. Attempting to Integrate Figurative Language Comprehension into the CHC Model

In recent years, there has been an interest in exploring the underlying cognitive processes involved in figurative language comprehension (Chiappe and Chiappe 2007; Pereira de Barros et al. 2010; Pierce and Chiappe 2009; Silvia and Beaty 2012). In this light, some studies suggest connecting the comprehension of figurative language to the CHC model of cognitive abilities.

Regarding the comprehension of figurative language, certain aspects of Glucksberg’s (2003) property attribution model can be adopted to conceptualize the cognitive mechanisms involved in figurative language comprehension. The formation and maintenance of a higher-order attributive category contains aspects of the concept of retrieval fluency (gr), which is described by Schneider and McGrew (2018) as “the rate and fluency at which individuals can produce and selectively and strategically retrieve verbal and nonverbal information or ideas stored in long-term memory” (p. 102). Tasks designed to assess retrieval ability typically require individuals to generate members of a given category based on a provided cue (e.g., list synonyms for the word “great”). In the context of Glucksberg’s (2003) model, the process of searching memory for a suitable vehicle (for example, “jail”) to attribute to a specific topic (in this example, “job”) bears resemblance to the selective retrieval processes associated with gr.

Furthermore, as pointed out above, a key aspect of Glucksberg’s (2003) attribution model is the process of relating two semantically unrelated concepts (e.g., “job” and “jail”) and requires identifying a hidden meaning which is similar to a rule (the rule being to relate to semantically unrelated concepts to each other). Within the CHC model, this ability is reflected in the narrow ability induction, which is the key component in fluid intelligence (Gf). Schneider and McGrew (2018) define induction as “the ability to observe a phenomenon and discover the underlying principles or rules that determine its behavior. This ability is also known as rule inference” (p. 93).

At the same time, it is necessary to prevent literal or adjectival information closely linked to the topic and vehicle from interfering with the goal of establishing a figurative connection (e.g., while some jobs can be a jail, they do not share the physical characteristics of a jail). We would expect fluid abilities to facilitate the search process by maintaining the task goal and delineating the abstracted meaning and/or inhibiting other inappropriate associations that compete for activation in memory (Gernsbacher et al. 2001). In this sense, it seems reasonable to posit that fluid intelligence contributes to the comprehension of figural language through induction. Thus, it is reasonable to assume that FLC might be part of gf. 

However, the comprehension of figurative language requires that the person has the relevant knowledge needed to identify the rules hidden in the word meanings. Such knowledge might be general vocabulary or lexical knowledge (Kan et al. 2011) but also specific language abilities. These abilities can be found in the CHC model, for example, in reading and writing (grw) and domain-specific knowledge (Drnyei and Skehan 2003; Li et al. 2015). All of these abilities are summarized under acquired knowledge in the CHC model. As mentioned earlier, when a person is familiar with a particular figurative expression, processing times for figurative and non-figurative utterances with similar meanings are comparable. This suggests that if the figurative meaning has been learned earlier, it becomes readily accessible, particularly for familiar examples of metaphors and ironies (Cronk and Schweigert 1992; Giora and Fein 1999; Keysar 1989). Thus, based on the processes described to underly the comprehension of figurative language, it is reasonable to allocate this ability not only within the nomological network of Gf but also acquired knowledge. Therefore, we will test these allocations in the current study to provide evidence supporting the position of figurative language comprehension in the CHC model. We will do so by testing a series of structural equation models in a head-to-head fashion. Thereby, we are following the example by Callis et al. (2023), who demonstrated the position of financial literacy in the nomological network of the CHC model by testing different theoretical assumptions in a head-to-head fashion. 

## 6. Considering Personality for the Comprehension of Figurative Language

As already suggested, when examining the comprehension of figurative language, it is important to consider not only the influence of linguistic and cognitive features but individual differences like personality. Figurative language serves specific pragmatic purposes relating to a person and their interaction with the environment and the people in it, including preserving social harmony, generating amusement, showcasing creativity, and fostering group cohesion (Dews and Winner 1995; Gerrig and Gibbs 1988; Gibbs 2000; Jorgensen et al. 1996; Roberts and Kreuz 1994). 

Similar to Ackerman’s (1996) PPIK model and Ziegler et al.’s (2012, 2015, 2018) openness-fluid-crystallized-intelligence (OFCI) model, the CHC model links fluid ability and knowledge with Openness. In particular, it is assumed that investing fluid ability increases knowledge (Cattell 1943, 1987). However, this investment is also influenced by a person’s willingness to engage with new stimuli, which is reflected in Openness. Consequently, more open people experience more learning situations, thereby training gf and acquiring more knowledge over time, which has a lasting impact throughout an individual’s lifetime (Ziegler et al. 2015). 

Thus, in the context of comprehending figurative language, individuals with a higher degree of Openness are inclined to actively engage in more reading and linguistic practice (Trapp and Ziegler 2019). This proactive involvement leads to enhanced fluid intelligence, which, in turn, could then contribute to a greater command of figurative language. Additionally, considering that creativity is a facet of Openness (DeYoung et al. 2012), individuals with greater Openness are more likely to exhibit heightened creativity in the generation and comprehension of figurative language. 

Some studies around personality and figurative language seem to support this notion. They have found significant relations between Openness and metaphor creativity. People who exhibit high Openness consider themselves creative. In addition, they show an interest in art and have creative hobbies (Conner and Silvia 2015). They also perform better in various creative tasks, such as divergent thinking (Silvia et al. 2009) and humor production (Nusbaum et al. 2017; Sutu et al. 2019). In general, research has predominantly demonstrated that the generation of creative metaphors incorporates cognitive functions like fluid intelligence as well as personality traits that facilitate creative thinking and creative problem solutions, such as Openness (Beaty and Silvia 2013; Pereira de Barros et al. 2010) and the need for cognition (Watts et al. 2017). Whether such a relation also occurs for figurative language comprehension will be tested here. 

## 7. Different Tests to Measure Figurative Language Comprehension

To explore the possibility of allocating FLC into the CHC model, sound measures are needed. The heterogeneous definitions and models for FLC have resulted in a heterogeneous landscape of tests. Such tests often include only one category of figurative language (mainly metaphors) and are conducted predominantly on clinical samples. The samples used are often small (*N* < 45) (Garcia-Albea and Gavilan 2009) and contain only clinical subjects and/or no healthy control group (e.g., Elvevåg et al. 2011; Gavilán and García-Albea 2011; Piovan et al. 2016), which limits ways to test their psychometric quality. The best known standardized diagnostic test for assessing proverb comprehension in the English-speaking world is Gorham’s Proverb Test (Gorham 1956), and it continues to be employed even in the present day (e.g., Knight et al. 2023). Barth and Küfferle (2001) developed a German proverb-metaphor test designed to assess the thinking patterns in patients suffering from schizophrenia or depression regarding figurative language comprehension, and the test has been frequently used in clinical trials (e.g., Brüne and Bodenstein 2005; Kircher et al. 2022; Leyhe et al. 2011; Uekermann et al. 2008). In this study, we will use three newly developed tests measuring figurative language comprehension. As mentioned above, the diverse definitions of figurative language comprehension contribute to a wide range of tests within this field. In the tests designed for figurative language comprehension thus far, numerous confounding factors were often overlooked. Additionally, certain tests were exclusively administered to clinical samples, neglecting an examination of figurative language comprehension in healthy subjects. In the recently developed items, refinements have been applied to both syntax and contextual presentation. Our primary focus has been on developing items tailored to assess the comprehension of proverbs.

## 8. The Present Study

In conclusion, figurative language comprehension is an ability relevant in everyday life. Discerning the psychological processes relevant for figurative language comprehension suggests similarities with other cognitive abilities, namely fluid intelligence and acquired knowledge and their narrower abilities in particular. These abilities seem of relevance as they pertain to the processing and decoding of new information and the knowledge needed for this, respectively. Thus, it seems reasonable to use the CHC taxonomy as a theoretical framework to test hypotheses regarding the position of figurative language comprehension amongst other human cognitive abilities (Schneider and McGrew 2018). Prior research has put a focus on diverse psychological processes and their role in figurative language comprehension rarely considering such a broad framework as a nomological network in which to anchor figurative language comprehension. The aim of this study is to investigate whether figurative language comprehension can be integrated in the CHC model of cognitive abilities, specifically gf, acquired knowledge (Schneider and McGrew 2018), or as its own factor under g. As a first step, we will test the items’ dimensionality, estimate the test score’s reliability, and look at evidence supporting the convergent validity with the Proverb–Metaphor Test (Barth and Küfferle 2001). These newly developed tests will then be used to allocate figurative language comprehension in the gf or acquired knowledge part of the CHC model as its own factor under g.

In light of the few studies conducted so far, we expect that the tests we developed measure a unidimensional variable: comprehension of figurative language. We have no strong prediction regarding the allocation of figurative language comprehension to gf or acquired knowledge, as both can be argued. The relation with Openness is also plausible but has not been established so far. Therefore, we will test both aspects in an explorative manner.

## 9. Methods

### Procedure

Participants were recruited by contacting schools and using mailing lists to include students from different schools and universities. The data were collected as part of the data collection for the validation of the Berlin Aptitude Test for Psychology (BSF-P) (Horstmann et al. 2023). All tests were administered via the formR survey platform (Arslan et al. 2020) in an online questionnaire. First, participants had to answer a standard set of questions regarding age, gender, and educational status. Then, participants completed the tests described under instruments. The tests were all administered in German, and it took about one hour in total to complete the tests. Participants had the possibility to request individual feedback on the personality questionnaire. The current study followed the Ethical Principles of Psychologists and Code of Conduct outlined by the American Psychological Association (APA). These guidelines were implemented to safeguard the rights and well-being of the participants.

## 10. Sample

Initially, the link to the questionnaire was opened 2629 times. As can be expected in an online survey, many individuals started the study but did not proceed to complete any question and opted out of the process entirely without any information given. These data sets as well as data sets from participants who had provided incomplete data (for example no session ID), making it impossible to merge their data sets, were excluded, which resulted in *N* = 1040. Furthermore, the data were controlled for duplicated data, and the duplicated data were deleted, resulting in a final sample of *N* = 909 participants who completed all relevant questionnaires. Of these, 60% (545) were women. The mean age was *M_age_* = 17.87 (*SD_age_* = 2.6, *median_age_* = 17). The sample was drawn from multiple schools and universities. Here, entire age cohorts in several schools were tested. The target population comprised German high school students in 11th or 12th grade (89.33%), participating for monetary compensation, and German undergraduate students in psychology (10.34%), participating for course credit. The remaining participants were in between educations or doing a voluntary social year. All participants were German natives or have lived in Germany from an early age. 

## 11. Instruments

Data from the following instruments were obtained from the participants:

### 11.1. Comprehension of Figurative Language

The test battery assessing figurative language comprehension (FLC) was developed by the Charité Berlin in the research group for psychotic disorders by Dr. Ivan Nenchev and Prof. Dr. Christiane Montag and was organized in three different tests: the Reverse Paraphrase Test (RPT), the Literal Paraphrase Test (LPT), and the Proverb Test (PT) (Nenchev and Montag 2023). Item construction was based on existing theoretical models and is intended to be mainly used for research targeting the general German public.

The RPT consists of 14 items and has a single-choice format. Participants were presented with a generic non-proverbial sentence and subsequently asked to select the most suitable proverb in accordance with it out of four choices. These choices contain different proverbs whereby only one of the proverbs is the correct interpretation of the generic non-proverbial sentence. The LPT consists of 20 items and has a single-choice format. Participants were presented with a literal sentence and were required to select the correctly rephrased synonymous literal sentence from a set of five answer options. In both tests, participants had to distance themselves from a sentence, to reformulate, and transform it. It involves moving away from a purely content-based consideration without completely disregarding grammatical rules. Ultimately, this skill goes beyond mere reading the ‘written word’ and understanding its meaning. It aligns with what Glucksberg (2003) describes as seeking higher-order attributive categories and ‘fitting’ them to the sentence.

The PT consists of 20 items and had an open answer format. Participants were asked to read a short text and to think of a proverb that would fit the text. The answers to the PT were rated by two expert raters. A strict approach was chosen in which the raters decided that only the target proverb or some slight variations (for example, correct answer: “Many roads lead to Rome”; slight variation, but still rated as correct: “All roads lead to Rome”) would score a point. When the raters had disagreements on the correct item answers, they discussed and came up with an agreed upon solution. The raters also established a criterion regarding spelling errors, which considered whether an answer would be considered correct in an oral examination scenario. In order to assess the inter-rater reliability of the ratings, Cohen’s kappa coefficients were estimated. The Cohen’s kappa coefficients were interpreted according to the guidelines proposed by Landis and Koch (1977). Cohen’s kappa ranged from κ = 1 to .65 depending on the item. Detailed information on each item can be found in Table S1 in the Supplementary Materials.

### 11.2. Item Example of the Reverse Paraphrase Test (RPT)

Instruction: Below you will see an abstract sentence. Then you are to decide which of the five proverbs best describes the abstract sentence in its meaning. Please mark the correct answer with a cross. Only one answer is correct at a time.


**Item**

**Translation**
Wenn die Aufsichtsperson nicht anwesend ist, verstoßen die anderen gegen die Regeln.If the supervisor is not present, the others violate the rules.a.Ein Hund, der nach zwei Hasen jagt, fängt keinen.A dog hunting for two rabbits catches none.b.
**Wenn der Hund schläft, hat der Wolf gut Schafe stehlen.**

**When the dog sleeps, the wolf has good sheep stealing.**
c.Wer den Hund füttert, dem leckt er die Hände.Whoever feeds the dog, the dog licks his hands.d.Zwei Hunde an einem Bein, kommen selten überein.Two dogs on one leg, rarely agree.e.Soll der Hund Schläge haben, so hat er Leder gefressen.If the dog is to have strokes, it has eaten leather.The correct answer is shown in bold.

### 11.3. Item Example of the Literal Paraphrase Test (LPT)

Instruction: In the next task, you are to mark which of the answer alternatives has the same meaning as the initial sentence. Please mark the correct answer with a cross. Only one answer is correct at a time.


**Item**

**Translation**
Das am Ende der Straße liegende Hotel war sehr teuer.The hotel located at the end of the street was very expensive.a.Das teure Hotel war am anderen Ende der Straße.The expensive hotel was at the other end of the street.b.
**Das Hotel, das am Ende der Straße liegt, war sehr teuer.**

**The hotel, which is located at the end of the street, was very expensive.**
c.Das Hotel war sehr teuer und lag am anderen Ende der Straße.The hotel was very expensive and was at the other end of the street.d.Das teure Hotel lag am anderen Ende der Straße.The expensive hotel was at the other end of the street.e.Das Hotel befand sich auf einer teuren Straße.The hotel was located on an expensive street.The correct answer is shown in bold.

### 11.4. Item Example of the Proverb Test (PT)

Instruction: Below you will be given some texts to read. For each text, you are to decide which proverb best fits the text. The texts are independent of each other and there are no wrong answers. Please write the proverb in the free space.


**Item**

**Translation**
Der Medienwissenschaftler Ben Bachmair untersuchte die Fernsehgewohnheiten von Kindern. In den Familien, wo die Eltern häufig vor dem Fernseher hocken, verbringen auch die Kinder mehrere Stunden vor dem Bildschirm. Die aktuelle Studie zeigte, dass es sich dann meist um männerdominierte Familien handelt,  in denen Actionfilme geguckt werden, was die Kinder, meist Jungen, übernehmen.Media scientist Ben Bachmair studied the television habits of children. In families where parents frequently sit in front of the TV, children also spend several hours in front of the screen. The current study showed that these are then mostly male-dominated families where action movies are watched, which the children, mostly boys, take over.
**Correct answer**

**Der Apfel fällt nicht weit vom Stamm.**

**The apple doesn’t fall far from the tree.**
The correct answer is shown in bold.

The most frequently used German language test for figurative language comprehension was also used. The Proverb–Metaphor Test by Barth and Küfferle (2001) consists of 19 metaphorical proverbs and has a single-choice format. Out of the total of 19 items, five items serve as dummy items and are excluded from score calculations. The primary application of this test lies within clinical settings, specifically in distinguishing the figurative language comprehension skills of individuals diagnosed with borderline personality disorder and schizophrenia. Since the test items were administered to mentally healthy individuals for the purpose of this study, the dummy items were omitted from the test battery. The remaining 14 items comprised the core components of the administered test. For each metaphorical proverb, the participants were given six possible interpretations from which the person had to choose the one that best explains the meaning of the proverb. 

### 11.5. Item Example of the Proverb–Metaphor Test (PMT)

Instruction: Below you will see one proverb at a time. You are to indicate what meaning the proverb has. Click on the correct answer alternative. There is always only one correct answer.


**Item**

**Translation**
Ist die Katze aus dem Haus, tanzen die Mäuse auf dem Tisch.When the cat’s away, the mice will play.a.Wenn keine Kontrollperson da ist, können die Mäuse machen, was sie wollen. If there is no control person, the mice can do whatever they want.b.Wenn Katzen und Mäuse nicht da sind, ist das Haus völlig leer.When cats and mice are not there, the house is completely empty.c.Wenn die Katze nicht da ist und aufpasst, kann man machen, was man will. When the cat is not around and paying attention, you can do whatever you want.d.
**Wenn keine Kontrollperson da ist, kann man machen, was man will.**

**If there is no control person, you can do whatever you want.**
e.Katzen fressen Mäuse. Mäuse können daher erst dann tanzen, wenn die Katze das Haus verlassen hat. Cats eat mice. Therefore, mice can dance only after the cat has left the house.f.Wenn niemand da ist, kann man alles alleine machen. When no one is around, you can do everything on your own.The correct answer is shown in bold.

### 11.6. CHC-Based Cognitive Abilities

Different cognitive abilities were measured using the Berlin Aptitude Test for Psychology (BSF-P; Horstmann et al. 2023). The BSF-P is a subject-specific study ability test based on the CHC model of cognitive abilities (Schneider and McGrew 2018). The BSF-P is used in student selection for the bachelor’s program in psychology in the university’s own selection process and is highly monitored. Based on a requirements analysis, results of which were located within the CHC model of cognitive abilities (Schneider and McGrew 2018), in order to theoretically anchor the requirements profile and to delineate the cognitive processes underlying the abilities, the BSF-P was constructed to cover six abilities: fluid reasoning (gf), divided into numerical (gfn), verbal (gfv) and figural reasoning (gff), reading and writing (grw), quantitative knowledge (gq), and domain-specific knowledge (i.e., knowledge of English, gkn). Grw, gkn, and gq together form acquired knowledge (ak), by which we followed the recommendation by Schneider and McGrew (2018) to not use these abilities as separate variables but as indicators of acquired knowledge. Furthermore, gfv, gfn and gff form fluid intelligence (gf). Using Rasch models, evidence for the dimensionality of each broad ability in the BSF-P has been provided (Horstmann et al. 2023). All subtests have been shown to adhere to the Rasch model and yield reliable and valid test score interpretations. The BSF-P was chosen deliberately, as it allows directly testing whether figurative language comprehension (FLC) is part of gf, acquired knowledge, or stands on its own. Our test selection aligns with the recommendations by Campbell and Fiske (1959) and Ziegler (2020) regarding construct validity evidence. In that sense, to allocate a measure within a nomological network, convergent and discriminant validity evidence are needed. Positioning FLC as part of either gf or acquired knowledge and testing these assumptions provides exactly this kind of evidence. If FLC belonged to gf, the model where FLC is allocated within the measurement model of gf should fit better than a model where FLC is allocated within the measurement model of acquired knowledge. This would support convergent and discriminant validity. If a model with FLC allocated to acquired knowledge fitted better, the opposite conclusion would have to be drawn. If FLC is an adjacent broad ability, neither belonging to the measurement model of gf nor acquired knowledge, a model where FLC directly loads the g factor above gf and acquired knowledge should fit best. The BSF-P allows capturing fluid abilities and what is called acquired knowledge in accordance with the most recent CHC model. As outlined above, acquired knowledge is operationalized by a test for reading and writing, one for domain-specific knowledge (English) and one for quantitative knowledge. As such, the operationalizations of gf and acquired knowledge are broad and, looking at the psychological processes captured, entail those which resemble the processes supposedly making up FLC. As such, the BSF-P provides a theoretically meaningful nomological network to test different allocations of FLC. 

### 11.7. Personality

To measure participants’ personality traits, we employed the German adaptation of the Big Five Inventory 2 (BFI-2; Danner et al. 2016). Using a five-point rating scale ranging from 1 (“*strongly disagree*”) to 5 (“*strongly agree*”), participants indicated the extent to which they agreed with each of the 60 items. Each of the five domains (Extraversion, Openness, Emotional Instability, Agreeableness, Conscientiousness) encompassed 12 items, four per facet.

### 11.8. Further Tests Not Used in the Current Study

Furthermore, there were other measurements conducted during the validation of the Berlin Aptitude Test for Psychology: the Achievement Motive Scale (AMS) (Lang and Fries 2006), the Achievement Goal Questionnaire (AGQ) (Elliot and McGregor 2001) and the O*NET IP Short Form (Rounds et al. 2010). Data for these tests were not used here. 

### 11.9. Planned Missing Data Design

To save resources during data collection, a *planned missing data design* (Graham et al. 2006; Little and Rubin 2020) was used for the items of the Literal Paraphrase Test (LPT), the Proverb Test (PT), the Proverb–Metaphor Test (PMT), and the BSF-P items. This approach is endorsed by Lawes et al. (2020) and has been shown to yield robust results, especially in factor-analytic designs (Revelle et al. 2017). For the Reverse Paraphrase Test (RPT), all participants received all 14 items.

Hence, five different test versions were specified, and each participant received one. Each version contained a mix of unique and shared items. In each test version, there were two unique items out of the Literal Paraphrase Test and the Proverb Test, one unique item from the Proverb–Metaphor Test, and from the BSF-P, there were three or six unique items depending on the subtest. Additionally, all test versions contained 10 shared items for the Proverb Test, 10 shared items for the Literal Paraphrase Test, and 9 shared items for the Proverb–Metaphor Test, and for the BSF-P, there were between 6 and 12 shared items depending on the subtest. These so-called ‘linking items’ were necessary in order to create a link between the test versions and allowed estimating missing information based on multiple imputation. Based on this, it was possible to perform further statistical calculations. 

## 12. Data Analysis

The data were analyzed in several steps. All materials can be found in the Online Supplementary Materials. 

### 12.1. Statistical Analysis

Statistical analyses were conducted using the software *R* (Version 4.2.3; R Core Team, 2023) and *R-Studio* (Version 2023.3.0.386; Posit team, 2023), especially the *missForest* package (Stekhoven and Bühlmann 2012) and the *lavaan* package (Rosseel 2012), as well as the *psych* package (Revelle 2023) and the *apaTables* package (Stanley 2021) were used.

Based on the *planned missing data design* (Graham et al. 2006; Lawes et al. 2020), the missing item answers in the Literal Paraphrase Test, the Proverb Test, the Proverb–Metaphor Test (Barth and Küfferle 2001) and the BSF-P were imputed using the package *missForest*, which is an iterative non-parametric random forest machine learning algorithm (Stekhoven and Bühlmann 2012). There were 40% of planned missing data with regard to the items of the Literal Paraphrase Test and 28.57% with regard to the items of the Proverb–Metaphor Test, as each participant received just one of five test versions. The shared items made it possible to create a link between all test versions in this missing completely at random multi-matrix design and then use multiple imputation to estimate missing values with a random forest classifier trained on the observed values of a data matrix to predict the missing values. Therefore, several replacement values for the missing data were imputed.

The study design did ensure that the missing values were missing completely at random (MAR; Zhang and Yu 2022). It has been shown that missing completely at random ensures that imputations are accurate and efficient (Lawes et al. 2020) and is particularly valuable in factor analytical approaches.

### 12.2. Structural Validity

In order to provide a better overview, the following steps describe the plan to test structural validity in detail.

### 12.3. CFAs for the Three Tests of Figurative Language Comprehension

In a first step, three CFAs were conducted separately to examine each FLC subtest’s measurement model (M_RPT_, M_LPT_, M_PT_) in the sample with the newly developed items as indicators to confirm that each test is unidimensional (Ziegler and Hagemann 2015). We randomly divided the dataset into two samples. In the first sample (*n* = 363), the measurement models were tested, and changes were made when necessary. In the second sample (*n* = 546), the models were confirmed. 

The CFA for the Proverb Test was performed on a sample of 450 participants to achieve sufficient model fit in the subsample for which data on cognitive ability were accessible. Data collection for the Proverb Test was terminated after the preliminary analysis indicated that a sufficient model fit could not be achieved. Additionally, the Proverb Test was found to be the most time-consuming assessment.

For all CFAs, the robust weighted least squares estimator (WLSMV) was used to account for the ordinal measurement level of the variables (Brauer et al. 2023). In addition, McDonald’s *ω* was estimated as a reliability estimate. Model fits for the three models were evaluated using the Chi-Square Goodness-of-Fit statistic, Comparative Fit Index (CFI; Bentler 1990), Root Mean Square Error of Approximation (RMSEA; Browne and Cudeck 1993), and Standardized Root Mean Squared Residual (SRMR; Bentler 1990). The following cut-offs indicated acceptable model fits: CFI > .90, RMSEA < .06, and SRMR < .08 (Hu and Bentler 1999; Kline 2005). In case of misfit, single items with poor psychometric quality (e.g., extremely large (*a* > .95) or negative loadings or extreme item difficulties) were dropped.

### 12.4. Theoretical Models for the Three Tests of Figurative Language Comprehension

In a second step, measurement models for the subtests were combined, and different latent structures were tested (M_gfm_, M_cfm_, M_bfm_). As described above, the Proverb Test (PT) had to be excluded at this stage.

We tested a general factor model (M_gfm_), a correlated factor model (M_cfm_), and a bifactor S-1 model (Mbfm; Eid et al. 2017). The general factor model proposed a model where the items of the two tests are indicators of a single latent factor, reflecting the idea of a shared ability, which is referred to as ‘figurative language comprehension’. 

In the correlated factor model, the items of the tests are influenced by test-specific but correlated factors, reflecting the idea of correlated yet distinct abilities. So, each of the two tests (RPT, LPT) would be measuring figurative language comprehension, but both would also capture specific aspects of the variability of the variable. 

The bifactor S-1 model was based on the assumption that the observed variables are influenced by a general factor (figurative language comprehension) and test-specific factors, reflecting the idea of a general ability and test-specific, nested abilities. This model would allow the separation of the joint influence of figurative language comprehension from the specific influences that affect only certain variables.

To determine if the collected data could be more accurately described by the general factor model, the correlated factor model, or the bifactor S-1 model, a statistical analysis was performed to compare the various model types. The models were assessed using confirmatory factor analyses (CFAs) with a robust maximum likelihood (MLR) estimator. The same thresholds indicating acceptable model fits were utilized.

To compare the models, we used the Chi-Square Goodness-of-Fit statistic and the difference in CFI (Meade et al. 2008). Moreover, the Akaike Information Criterion (AIC) values (Akaike 1974) were compared. The model with the lowest overall information criteria was selected. Furthermore, McDonald’s *ω* was estimated.

### 12.5. Comparison of Different Models for the Allocation of Figurative Language Comprehension

In a third step, the preferred model from the previous step was combined with the structural model of the BSF-P. 

Three different models were tested, each providing a different allocation of figurative language comprehension within the CHC model of cognitive abilities. Testing of the models took place in a reduced data set, since not all participants who completed the tests on figurative language comprehension also completed the BSF-P. So, both data sets were combined, leaving 450 participants who completed the tests on figurative language comprehension and the BSF-P. 

**Figure 1:**
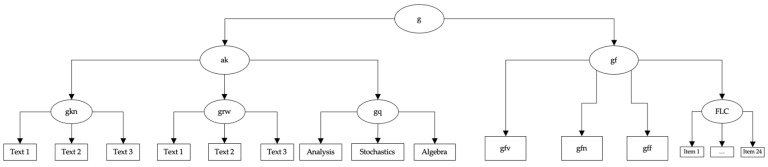
Graphic representation of the allocation of figurative language comprehension as part of fluid intelligence (gf); g factor (g), acquired knowledge (ak), English proficiency (gkn), reading comprehension (grw), quantitative knowledge (gq), verbal reasoning (gfv), numerical reasoning (gfn), figural reasoning (gff), figurative language comprehension (FLC).

**Figure 2:**
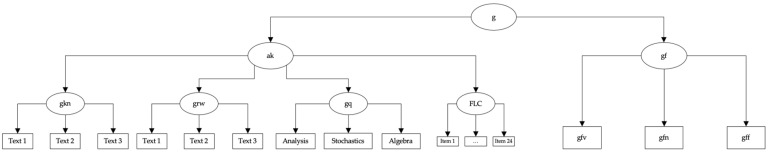
Graphic representation of the allocation of figurative language comprehension as part of acquired knowledge (ak); g factor (g), fluid intelligence (gf), English proficiency (gkn), reading comprehension (grw), quantitative knowledge (gq), verbal reasoning (gfv), numerical reasoning (gfn), figural reasoning (gff), figurative language comprehension (FLC).

**Figure 3:**
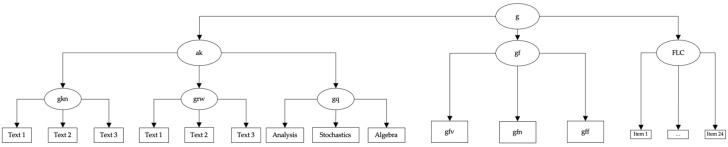
Graphic representation of the allocation of figurative language comprehension as a factor under the g factor (g); acquired knowledge (ak), fluid intelligence (gf), English proficiency (gkn), reading comprehension (grw), quantitative knowledge (gq), verbal reasoning (gfv), numerical reasoning (gfn), figural reasoning (gff), figurative language comprehension (FLC).

The first model tested if figurative language comprehension could be allocated in the measurement model of fluid intelligence (FLC-gf). The second model tested if figurative language comprehension could be allocated as a part of acquired knowledge (FLC-ak). Finally, the third model tested the allocation of figurative language comprehension as an independent factor under the g-factor (FLC-g). Confirmatory factor analyses (CFAs) with a robust maximum likelihood (MLR) estimator were used, the same thresholds indicating acceptable model fits were utilized. The Chi-Square Goodness-of-Fit statistic and the difference in CFI (Meade et al. 2008) were used to compare the models. The Akaike Information Criterion (AIC) values (Akaike 1974) were compared. The BSF-P data were modeled as described in Horstmann et al. (2023). Figure 1, Figure 2 and Figure 3 depict the different theoretical models as well as the entailed measurement models.

### 12.6. Convergent and Discriminant Validity and Reliability Evidence

Evidence for convergent and discriminant validity will be gathered. For convergent validity, the data collected by the Proverb–Metaphor Test (Barth and Küfferle 2001) were used. Here, we expect a substantial correlation with our figurative language comprehension scores.

In order to explore the nomological network surrounding figurative language comprehension, our investigation will examine its relation with the personality trait of Openness. Previous research has established connections between personality factors and figurative language. To be able to better gauge the size of the relation between figurative language comprehension and Openness, we will investigate all five personality traits. Specifically, we anticipate observing the strongest correlations between figurative language comprehension and Openness. 

## 13. Results

### 13.1. Descriptive Statistics

The descriptive statistics for each test, utilizing sum scores, can be found in Table 1. Notably, the statistics in the table reflect the sum scores after the exclusion of items that exhibited misfit during subsequent analysis. A comprehensive description of the process and criteria for excluding these items is provided in the following paragraph. Analysis of the item statistics indicates that participants found the items relatively easy to solve. For more detailed item-level statistics, including statistical information pertaining to each individual item, please refer to Table S2 in the Supplementary Materials.

### 13.2. Measurement Models for the Three Tests of Figurative Language Comprehension

Initially, the analysis included three newly developed tests (Reverse Paraphrase Test, RPT; Literal Paraphrase Test, LPT; Proverb Test, PT) along with a total of 54 items. To ensure the unidimensionality of items within each subtest, we conducted an examination of the three measurement models. To this end, we first divided the dataset into two samples. In one sample (*n* = 363), we tested, and where needed adapted, our measurement models; in the second data sample (*n* = 546), we confirmed our models. The RPT demonstrated an immediate sufficient fit in the first sample without the need to make any adjustments, whereas the LPT and PT did not meet the required fit criteria in the first sample when considering all items.

Based on the predefined criteria above, several items were excluded from the LPT. Nine items were eliminated due to extremely large loadings (*a* > .95) in combination with extreme item difficulties, while one item was dropped due to a negative loading.

Following these adaptations, the model for the LPT was again tested in the first, smaller sample and then tested in the second sample. The model fit was found to be sufficient, as indicated in Table 2, demonstrating unidimensionality. The item loadings for the RPT ranged from .596 to .841, and for the LPT, they ranged from .697 to .944. For a comprehensive overview of the item loadings for each test, please refer to Table S3 in the Supplementary Materials.

The PT, or Proverb Test, presented the most challenges. When examining the initial model fit and applying the same criteria as for the RPT and LPT, no satisfactory model fit could be achieved. Consequently, an exploratory factor analysis was conducted; however, no meaningful solution could be derived. As a result, the Proverb Test was excluded from further data collection and analysis. Therefore, to explore the internal structure of the different operationalizations for figurative language comprehension, only the RPT and LPT were retained for subsequent analyses.

### 13.3. Theoretical Models of Figurative Language Comprehension

After the exclusion of the Proverb Test and the exclusion of ten items from the LPT, an evaluation of different theoretical models underlying the relation between the Reverse Paraphrase Test (RPT) and the Literal Paraphrase Test (LPT) was conducted. We examined three different models outlined above: a general factor model (M_gfm_), a correlated factor model (M_cfm_), and a bifactor S-1 model with a general factor and nested test-specific factors (M_bfm_). These analyses were conducted in a dataset consisting of all 909 participants. The model fit indices are presented in Table 3, demonstrating good fit for all models. The correlated factor model exhibited a good fit among the tested models. The correlation between the two latent factors in the correlated factor model was high (*r* = .847), suggesting substantial conceptual overlap. The bifactor S-1 factor model exhibited the best fit among the tested models. In addition, considering not only the model fit measures but also the Akaike Information Criterion (AIC), the bifactor S-1 model was selected as the most suitable model for further analyses. Notably, the RPT served as the reference method in this model. Item loadings in the bifactor S-1 model ranged from .327 to .742 for the general factor. Further details regarding item loadings can be found in Table S4 in the Supplementary Materials.

### 13.4. Comparison of Different Theoretical Models for the Allocation of Figurative Language Comprehension in the CHC Model

Three distinct models were examined to explore the allocation of figurative language comprehension within the CHC model. Each model presented a different approach to incorporating figurative language comprehension within the CHC framework. The results, as displayed in Table 4, indicated that the model treating figurative language comprehension as a related, yet distinct ability (FLC-g) exhibited the best fit. The AIC also favored the FLC-g model. Consequently, the FLC-g model was chosen as the preferred one.

The loading of the g factor on FLC was .326. The loading of g on gf was .813 and the loadings of g on acquired knowledge (formed out of grw, gkn and gq) ranged from .847 to .992, respectively. Detailed item loadings for all three models can be found in Table S5 in the Supplementary Materials. 

### 13.5. Correlations with Personality

To explore the nomological network, correlations with scores for the convergent measure, the Proverb–Metaphor Test (PMT), and the personality measurement (BFI-2) were estimated. Pearson correlations were used. The results revealed significant positive correlations among the scores of the newly developed tests, the Reverse Paraphrase Test (RPT), and the Literal Paraphrase Test (LPT; *r* = .72, *p* < .01), as well as between the RPT and the PMT scores (*r* = .69, *p* < .01), and the LPT and the PMT scores (*r* = .75, *p* < .01). These correlations provide evidence for the convergent validity of the RPT and LPT scores.

In addition, correlations with Openness scores were examined in the present study. The results showed significant positive correlations between the Openness domain score and the RPT score (*r* = .24, *p* < .01), the LPT score (*r* = .19, *p* < .01), and the PMT score (*r* = .24, *p* < .01). Furthermore, significant positive correlations were found between the Openness facet scores for Intellectual Curiosity and the scores for RPT (*r* = .30, *p* < .01), LPT (*r* = .23, *p* < .01), and PMT (*r* = .26, *p* < .01), between the facet Aesthetic Sensitivity score and the scores for RPT (*r* = .19, *p* < .01), LPT (*r* = .16, *p* < .01), and PMT (*r* = .22, *p* < .01), and between the facet score for Creative Imagination and the scores for RPT (*r* = .07, *p* < .05) and the PMT (*r* = .08, *p* < .05). Correlations of the factor scores of figurative language comprehension with the Openness domain and facet scores were also examined; no correlation was significant. 

Finally, the possibility of the relation between figurative language comprehension and Openness being influenced by fluid intelligence and/or acquired knowledge led to an examination to determine whether the existing correlations persisted when accounting for the influence of acquired knowledge and fluid intelligence. This process entailed utilizing partial correlation analysis to identify significant correlations and evaluate potential incremental effects. Correlations between the Openness facet scores for Intellectual Curiosity and the scores for RPT (*r* = .24, *p* < .01) and for LPT (*r* = .19, *p* < .01) were smaller but still significant. Correlations with factor scores and partial correlations were drawn from a sample of 450 people, resulting from the participants who completed both the tests on figurative language comprehension and the BSF-P.

## 14. Discussion

This special issue aims at exploring the interplay of personality and intelligence, a research area which has attracted more and more attention in recent years (Colom et al. 2019). In this study, we were interested in figurative language comprehension (FLC), which could be another ability profiting from an interplay between cognitive abilities and personality. 

Although FLC has so far not been directly considered as a cognitive ability in common frameworks like the CHC model, there is theory and empirical evidence suggesting such an allocation. It seems reasonable to assume that the comprehension of figurative language requires some level of cognitive ability to understand and appropriately extract the metaphorical and symbolic meanings. On the other hand, there is also evidence that the comprehension of figurative language may be related to personality traits. Certain personality traits, such as Openness, might be associated with an increased tendency to use and be met by figurative language. Individuals with a strong preference for figurative forms of expression could possibly be more attracted to metaphors, proverbs, and symbolic forms of language. Likewise, higher Openness is likely to bring more contact with such stimuli by more reading activity (Trapp et al. 2019; Trapp and Ziegler 2019). 

To follow up on these ideas, we explored the relation between figurative language comprehension, major components of the CHC model of cognitive abilities and the personality trait Openness as well as its facets Intellectual Curiosity, Aesthetic Sensitivity, and Creative Imagination.

The aim of this study was to investigate whether the ability to comprehend figurative language can be allocated in the CHC model of cognitive abilities (Schneider and McGrew 2018) and therefore extend the CHC model. We expected that the tests we developed indeed were able to measure figurative language comprehension. This expectation was supported. Regarding our research question addressing where to position FLC in the CHC model, the results tentatively suggested an allocation as a broad ability, which will be discussed below. 

### 14.1. Psychometric Quality of Different Figurative Language Comprehension Operationalizations

For the comprehension of figurative language, three newly developed tests and one established test (Proverb–Metaphor Test; Barth and Küfferle 2001) were used. The tests were applied together with other measures in a large sample, consisting of students and pupils. Descriptive analysis revealed that the items were rather easy to solve for the participants. Looking at the measurement model of each of the three tests separately showed that two tests (Reverse Paraphrase Test, RPT; Literal Paraphrase Test, LPT) yielded a good fit after the exclusions of items within the Literal Paraphrase Test, supporting the notion that each test is unidimensional. Both test scores showed sufficient reliability using McDonald’s *ω*. The third test, the Proverb Test, did not reach a good fit even after the exclusion of numerous items. An explorative approach was tried as well but revealed no clear structure underlying the data. It was evident that many respondents could not think of a proverb to go with the stimulus text, as the missing values were prominent. The items might have been too far out of the participant’s experience. Therefore, the test was dropped from further analysis.

Evidence for construct validity of the RPT and LPT was collected as well. To assess convergent validity, we utilized data from the Proverb–Metaphor Test (PMT; Barth and Küfferle 2001), and we anticipated high correlations with the figurative language comprehension scores of the RPT and LPT. The present results provide evidence supporting the validity of the test score interpretation of the newly developed tests, RPT and LPT, as a significant positive correlation was found between the scores of both tests and the PMT scores. This indicates that our measures seem to capture similar constructs related to figurative language comprehension.

The models for the RPT and the LPT were combined, and different latent structures were tested (M_gfm_, M_cfm_, M_bfm_). Each model encapsulated a different theoretical idea. Whereas the general factor model (M_gfm_) reflected the idea of a shared ability, referred to as ‘figurative language comprehension’, the correlated factor model (M_cfm_) reflected the idea that each subtest captures a distinct ability. Finally, the bifactor S-1 model (M_bfm_) resembles Spearman’s idea of a general ability factor, which is accompanied by test-specific abilities. Our results revealed that both the correlated factor model and the bifactor S-1 model exhibited good fit, but the bifactor S-1 model had a slightly better fit. Considering the strong latent correlation in the correlated factor model along with the Akaike Information Criterion (AIC), and the theoretical underpinnings of the construct, it was determined that the bifactor S-1 model provided the best representation of the data. In this model, the Reverse Paraphrase Test (RPT) and the Literal Paraphrase Test (LPT) captured a common ability while also accounting for additional test-specific variance. It is noteworthy that the items in the LPT and RPT demonstrated some similarities. The RPT required respondents to ‘translate’ a generic non-proverbial (or non-figurative) sentence into the most suitable proverb in accordance with it, while the LPT involved the abstraction and the rephrasing of a non-figurative sentence. Importantly, despite these differences in the concrete composition of psychological processes, the figurative language comprehension tests for which measurement models could be found all load on one common factor. This shared factor does not solely represent language comprehension. If it did, a substantial correlation with our acquired knowledge factor, encompassing aspects of language comprehension, reading, and writing, would have been observed. However, empirical evidence does not support this. Moreover, a model including this common factor as a component of acquired knowledge does not fit the data best. Instead, our findings suggest that the focus of the construct under investigation is different, and we propose that the central marker might be an ability to abstract meaning from a non-figurative sentence and find fitting pictorial description, which is distinct from simple language comprehension. Thus, although both tests assessed the same underlying ability, they measured it in distinct ways. This breadth in psychological processes inherent in the tasks provides evidence for content validity. 

### 14.2. Allocating FLC in the CHC Model of Cognitive Abilities

Following up on the studies conducted on figurative language comprehension until now, we argued that the construct could be positioned within a nomological network of cognitive abilities. This perspective is supported by researchers who argue that figurative language comprehension requires the same fundamental mechanisms of language processing and comprehension as non-figurative language but adds further psychological processes (Gibbs 1994; Gibbs and Colston 2012; Glucksberg 2003).

When comparing different possible allocations of FLC within the CHC framework, the best fitting model assumed FLC to be a cognitive ability related yet distinct from fluid intelligence and gc. This model would suggest that despite relations with fluid intelligence and knowledge, FLC requires an additional, distinct set of psychological processes. As already outlined, we chose the g factor model as our preferred model, which suggests that FLC shares psychological processes with other cognitive abilities operationalized here, but it also comprises unique processes turning it into a potential additional broad ability in the CHC model.

Based on previous research, we had suggested above that figurative language comprehension could be conceived as part of acquired knowledge, such as general vocabulary, lexical knowledge, domain-specific knowledge, and reading ability (Drnyei and Skehan 2003; Kan et al. 2011; Li et al. 2015). The current results did not support this notion. It is worth noting that the operationalization of acquired knowledge in the BSF-P was broad but not comprehensive. For example, while the BSF-P included reading and writing ability, domain-specific knowledge (English as a foreign language), and quantitative knowledge, it did not include vocabulary, which is a prominent marker of crystallized intelligence (Schipolowski et al. 2014). In studies that have found evidence of a relation between figurative language comprehension and acquired knowledge (e.g., Giora and Fein 1999), researchers often used operationalizations, which included a wide range of declarative and procedural knowledge, language skills, and general knowledge acquired through experience, learning, and acculturation. These studies also revealed that the linguistic characteristics of figurative language play a significant role in its processing (Cronk and Schweigert 1992; Keysar 1989). Moreover, the linguistic context surrounding figurative language, including negation, quantifiers, and previous instances of figurative language within the same text, influences processing speed (Filik and Moxey 2010; Giora et al. 1998, 2007). It is also important to consider the content of domain-specific knowledge, in understanding figurative language, especially among adult participants. Ackerman and colleagues (Ackerman 1987, 1996, 2000; Ackerman and Heggestad 1997; Ackerman and Rolfhus 1999; Beier and Ackerman 2005; Rolfhus and Ackerman 1996, 1999) have extensively studied the domain of gkn in adults (also see Rusche and Ziegler (2023) for an example in German culture). Their research has demonstrated that learning new domain-specific knowledge, including declarative knowledge, is influenced not only by cognitive abilities but also by situational and individual interests as well as personality characteristics such as Openness and intellectual engagement. Ackerman’s (1996) intelligence-as-process, personality, interest, and intelligence-as-knowledge (PPIK) theory, as well as Ziegler et al.’s (2012, 2015) OFCI model, provide comprehensive explanations of the development of domain-specific knowledge. It is noteworthy that domain-specific knowledge differs from general and lexical knowledge in terms of stability in adulthood. While general and lexical knowledge tend to remain stable throughout adulthood, domain-specific knowledge (gkn), defined as expertise acquired through specialized training or professional practice, can continue to expand and evolve (Ackerman 2000; Ackerman and Rolfhus 1999). Considering figurative language comprehension, differences in comprehension abilities may be influenced by variations in domain-specific knowledge in the area addressed by the figure of speech to be deciphered. These differences in domain-specific knowledge could arise from individual differences in the willingness to accumulate more knowledge as adults, reflecting their intellectual curiosity or personality traits in addition to cognitive abilities. Thus, it could be argued that an even broader operationalization might yield different results. However, as Schipolowski et al. (2014) could demonstrate, correlations between measures capturing different aspects of acquired knowledge (back then the umbrella term crystallized intelligence was used; the term acquired knowledge was introduced in the 2018 CHC model revision) are typically around .6 or higher. Thus, it seems unlikely that a broader operationalization which would maximize the core variance of acquired knowledge would increase the loading of FLC. A different idea would be to match the content of the items in an FLC test with the interests of a person, assuming that a match between interests and content facilitates knowledge acquisition (Rusche and Ziegler 2022; Zhang and Ziegler 2022; Ziegler et al. 2018). Future research could test whether such conditions yield larger relations between FLC and acquired knowledge. 

Another perspective outlined above is the allocation of figurative language comprehension as part of fluid intelligence. Previous studies have established connections between fluid intelligence and the capacity to comprehend and generate creative metaphors (Beaty and Silvia 2013). These studies suggest that fluid intelligence plays a role in generating new figurative language and moderating the intrusion of abstract meanings when decoding figurative impressions (Silvia 2015). This aligns with certain aspects of Glucksberg’s (2003) property attribution model. When searching memory for a suitable vehicle (e.g., “jail”) to attribute to a specific topic (e.g., “job”), it becomes necessary to prevent literal or adjectival information closely associated with the topic and vehicle from interfering with the goal of establishing a figurative connection. For example, while some jobs might be metaphorically describable as a jail, they do not share the physical characteristics of an actual jail. Thus, fluid intelligence could play a role in the comprehension of figurative language. The current data do not support the idea that FLC is part of a gf measurement model. 

In the recent CHC revision (Schneider and McGrew 2018), the authors suggest a complex model of chained processes starting at more basic abilities (e.g., working memory) to more complex abilities (e.g., fluid intelligence) to end with acquired knowledge. Importantly, other personality traits, such as Openness or interests, are also included as suggested by models like the PPIK or OFCI. In a similar way, it seems reasonable to assume that FLC could be the product of an equally complex interplay of cognitive abilities and other personality traits. One ability not covered in the present research is creativity. Creativity, often linked to the generation of new figurative language, is also considered an ability associated with high levels of fluid intelligence, gc and retrieval fluency (gr) (Silvia 2015; Schneider and McGrew 2018). Moreover, possessing a substantial amount of well-organized domain-specific knowledge (gkn) has also been found to be associated with creativity (Weisberg 2006). Thus, future research should explore the relation of FLC with creativity as an ability. 

Another potential ability not operationalized here is retrieval fluency. Retrieval fluency (gr) can be defined as “the rate and fluency at which individuals can produce and selectively and strategically retrieve verbal and nonverbal information or ideas stored in long-term memory” (Schneider and McGrew 2018, p. 102). Throughout our daily lives, we are constantly required to retrieve facts, recall past events, remember names of acquaintances, and access other important information. The ability to retrieve this information, often without external cues, is crucial for the successful completion of many everyday tasks. Therefore, strategic retrieval processes are fundamental components of the overall cognitive system (Unsworth 2017). Although retrieval fluency is not explicitly included in the BSF-P, it is possible that figurative language comprehension is related to this cognitive process and may play an important role in understanding figurative language.

To sum up, the allocation of figurative language comprehension within the CHC framework as an additional broad ability beneath the g-factor received the strongest support from the current data. With retrieval ability or creativity, at least two other cognitive abilities will have to be investigated to solidify this hypothesis. Moreover, considering the psychological processes involved in FLC suggests that in order to develop FLC, an integrated framework is reasonable. Such a framework should encompass cognitive abilities like retrieval fluency (gr) and rule induction (gf), which are relevant to decipher figurative language or to retrieve knowledge enabling such an understanding (Schneider and McGrew 2018). Additionally, it should account for acquired knowledge, including general vocabulary, lexical knowledge, and domain-specific knowledge (Drnyei and Skehan 2003; Kan et al. 2011; Li et al. 2015). Also, reading ability (grw) plays a crucial role when encountering figurative language in textual contexts. By testing such an integrated approach in a longitudinal design, researchers could gain a deeper understanding of the regularities and complexities observed in figurative language comprehension (Gibbs and Colston 2012). However, despite these strong ties to cognitive abilities, integrative models like the PPIK or OFCI would also suggest that individual difference variables like Openness are crucial for our understanding of differences in FLC.

### 14.3. How does Figurative Language Comprehension relate to Personality?

Coming back to the complex framework of characteristics potentially relevant to explain FLC, the current results, in line with process models like the PPIK or OFCI model, suggest that certain personality traits also have to be included. Specifically, we expected to observe correlations between Openness and its facets with figurative language comprehension. The analysis revealed significant correlations between Openness and the newly developed tests (RPT and LPT) and the well-known PMT by Barth and Küfferle (2001) that measure figurative language comprehension. Notably, the highest correlations were observed with the Openness facet Intellectual Curiosity and Aesthetic Sensitivity across all three tests (RPT, LPT, PMT), while other correlations were negligible. This finding aligns with the environmental enrichment hypothesis, which is a fundamental part of the OFCI model (Ziegler et al. 2012, 2015). This hypothesis proposes that higher scores on Openness leads to more learning opportunities and provides the energy to engage with new stimuli (Ziegler et al. 2018). In particular, reading has been shown to be an activity relevant in environmental enrichment. Hence, the facet correlations, and specifically the items within the facets, suggest that higher scores on Aesthetic Sensitivity lead to more reading of poetry and literature. Since literature and poetry often employ figurative language, individuals with a greater interest in these domains are more likely to be exposed to and familiarize themselves with figurative expressions. Higher scores on Intellectual Curiosity in turn lead to more thinking about such new content. Further applying Cattell’s investment theory (Cattell 1943, 1987), which suggests that investing fluid ability increases knowledge, such activities would further the accumulation of figurative language comprehension. Importantly, whereas prior work has focused on how Openness and fluid ability contribute to the accumulation of knowledge, the current results insinuate that environmental enrichment and investment of fluid ability could also contribute to the development of FLC. Thus, the complex framework relevant to explain FLC most likely also includes Openness and its facets. 

### 14.4. Limitations and Further Research

The present study has several limitations that should be considered. First, the difficulty level of the items may have been too easy, which would lead to variances being low, and therefore, all relations found here might be lower bound estimates. It is important to note that the tests were initially developed for testing the comprehension of figurative language in a sample of schizophrenic patients. This could explain why our non-clinical sample had a high probability of solving the items correctly. Future studies should adjust the difficulty of the items to provide a more accurate measure of figurative language comprehension for healthy individuals. Furthermore, despite a certain breadth of tests, potentially relevant abilities within the CHC model should be revisited and a more comprehensive assessment conducted. It is possible that figurative language comprehension could be better integrated in another domain of the model and a more comprehensive model would reveal that. Further research should, for example, also cover Gr. This consideration applies equally to the assessment of figurative language comprehension. While three tests were employed to assess the comprehension, the exclusion of one complete test (PT) and several items from the LPT due to psychometric difficulties indicates a compelling need for a more comprehensive and diverse assessment of figurative language comprehension. 

Additionally, it is important to note that the test items used to assess figurative language comprehension possess cultural sensitivity and are specifically applicable to the German population. Caution should be exercised when generalizing the findings to other cultural contexts or populations, as the appropriateness and validity of these items may vary. In line with this, the sample drawn should also be considered with caution, as it is certainly restricted in age and education level. To summarize, this initial indication serves as a pivotal starting point, necessitating further research to inspire future investigations into the matter.

### 14.5. Conclusions and Outlook

This study contributes to a better understanding of the psychological process(ses) underlying figurative language comprehension. The current results provide a preliminary indication of the allocation of figurative language comprehension in the CHC model, and its relation with Openness suggests a framework of a complex interplay between personality and cognitive abilities, which might be at play to accumulate FLC. Overall, this suggests that figurative language comprehension might be the result of both cognitive abilities and personality traits, specifically investment traits (Von Stumm and Ackerman 2013). In that, FLC is similar to a competence which is defined as the result of a combination of non-cognitive traits and cognitive abilities (Blömeke et al. 2015). It is likely that both individual differences in cognitive abilities and personality may have an impact on the comprehension of figurative language and the development of this ability. As such, the current work stands as a first example which can inspire future research in this area to focus more on the functional integration of relations between intelligence, personality, and figurative language.

## Figures and Tables

**Table 1:** Means, standard deviations, and correlations with confidence interval.

Variable	M	SD	1	2	3	4	5	6	7	8	9	10	11	12	13	14	15	16	17	18	19	20	21	22
1.RPT	10.92	3.20																						
2.LPT	8.89	2.07	.72 **																					
			[.69, .75]																					
3.PMT	11.63	3.66	.69 **	.75 **																				
			[.65, .72]	[.72, .77]																				
4.Extraversion	3.49	0.67	.00	.02	.02																			
			[−.06, .07]	[−.04, .09]	[−.05, .08]																			
5.Agreeableness	3.82	0.56	.03	.08 *	.08 *	.14 **																		
			[−.04, .09]	[.01, .14]	[.01, .14]	[.08, .20]																		
6.Conscientiousness	3.48	0.68	−.10 **	−.03	−.01	.28 **	.30 **																	
			[−.16, −.04]	[−.09, .04]	[−.07, .06]	[.22, .34]	[.24, .36]																	
7.Negative Emotionality	2.81	0.70	.02	.02	.04	−.30 **	−.31 **	−.34 **																
			[−.04, .09]	[−.05, .08]	[−.02, .11]	[−.35, −.24]	[−.37, −.25]	[−.40, −.29]																
8.Openness	3.64	0.69	.24 **	.19 **	.24 **	.30 **	.19 **	.07 *	−.05															
			[.17, .30]	[.13, .25]	[.18, .30]	[.24, .36]	[.12, .25]	[.00, .13]	[−.12, .01]															
9.E_Sociability	3.36	0.90	−.06	−.03	−.03	.86 **	.09 **	.12 **	−.20 **	.14 **														
			[−.12, .01]	[−.09, .04]	[−.10, .03]	[.84, .88]	[.03, .15]	[.06, .19]	[−.27, −.14]	[.08, .21]														
10.A_Compassion	4.04	0.72	.08 *	.14 **	.15 **	.18 **	.82 **	.26 **	−.09 **	.21 **	.12 **													
			[.02, .14]	[.08, .20]	[.08, .21]	[.12, .24]	[.80, .84]	[.20, .32]	[−.16, −.03]	[.15, .28]	[.06, .18]													
11.C_ Organization	3.62	0.98	−.14 **	−.05	−.04	.14 **	.18 **	.84 **	−.16 **	−.03	.05	.15 **												
			[−.20, −.07]	[−.12, .01]	[−.10, .03]	[.08, .20]	[.11, .24]	[.82, .86]	[−.23, −.10]	[−.10, .03]	[−.01, .12]	[.09, .21]												
12.N_Anxiety	3.11	0.77	.03	.05	.07 *	−.26 **	−.11 **	−.19 **	.85 **	−.01	−.19 **	.07 *	−.06											
			[−.04, .09]	[−.01, .12]	[.01, .13]	[−.31, −.19]	[−.17, −.05]	[−.25, −.13]	[.83, .87]	[−.08, .05]	[−.26, −.13]	[.00, .13]	[−.12, .01]											
13.O_Aesthetic Sensitivity	3.43	1.05	.19 **	.16 **	.22 **	.13 **	.19 **	.04	.05	.84 **	.05	.23 **	.01	.09 **										
			[.13, .25]	[.10, .22]	[.16, .29]	[.07, .20]	[.12, .25]	[−.02, .11]	[−.01, .12]	[.81, .85]	[−.02, .11]	[.16, .29]	[−.06, .07]	[.02, .15]										
14.E_Assertiveness	3.53	0.84	.06	.04	.04	.79 **	−.05	.27 **	−.23 **	.26 **	.49 **	.04	.14 **	−.25 **	.08 *									
			[−.01, .12]	[−.03, .10]	[−.03, .10]	[.76, .81]	[−.11, .02]	[.20, .32]	[−.29, −.17]	[.20, .32]	[.44, .54]	[−.02, .11]	[.07, .20]	[−.31, −.19]	[.01, .14]									
15.A_Respectfulness	4.17	0.64	.00	.05	.04	.10 **	.80 **	.39 **	−.37 **	.14 **	.03	.56 **	.25 **	−.15 **	.13 **	.00								
			[−.06, .07]	[−.01, .12]	[−.02, .11]	[.04, .17]	[.78, .83]	[.33, .45]	[−.42, −.31]	[.08, .20]	[−.03, .10]	[.52, .61]	[.19, .31]	[−.22, −.09]	[.06, .19]	[−.06, .07]								
16.C_Productiveness	3.18	0.83	−.06	−.03	−.01	.32 **	.26 **	.85 **	−.37 **	.13 **	.17 **	.21 **	.51 **	−.24 **	.05	.28 **	.30 **							
			[−.13, .00]	[−.10, .03]	[−.07, .06]	[.26, .38]	[.19, .32]	[.83, .86]	[−.43, −.32]	[.06, .19]	[.11, .24]	[.14, .27]	[.46, .56]	[−.30, −.17]	[−.01, .12]	[.22, .34]	[.24, .36]							
17.N_Depression	2.54	0.90	.06	.04	.07 *	−.44 **	−.29 **	−.38 **	.83 **	−.05	−.36 **	−.14 **	−.22 **	.57 **	.05	−.28 **	−.29 **	−.40 **						
			[−.00, .13]	[−.02, .11]	[.00, .13]	[−.49, −.39]	[−.35, −.23]	[−.43, −.32]	[.81, .85]	[−.12, .01]	[−.42, −.31]	[−.20, −.07]	[−.28, −.15]	[.53, .62]	[−.02, .11]	[−.34, −.22]	[−.35, −.23]	[−.45, −.34]						
18.O_Intellectual Curiosity	3.83	0.78	.30 **	.23 **	.26 **	.28 **	.10 **	.05	−.10 **	.76 **	.12 **	.13 **	−.06	−.08 *	.44 **	.28 **	.08 *	.15 **	−.06					
			[.24, .35]	[.17, .29]	[.20, .32]	[.22, .34]	[.04, .16]	[−.01, .12]	[−.16, −.03]	[.73, .79]	[.06, .19]	[.07, .19]	[−.12, .01]	[−.15, −.02]	[.39, .49]	[.22, .34]	[.02, .15]	[.08, .21]	[−.13, .00]					
19.E_Energy Level	3.58	0.74	.02	.06	.05	.80 **	.33 **	.31 **	−.30 **	.36 **	.58 **	.30 **	.16 **	−.18 **	.22 **	.42 **	.25 **	.36 **	−.45 **	.30 **				
			[−.05, .08]	[−.00, .13]	[−.01, .12]	[.77, .82]	[.27, .39]	[.25, .37]	[−.36, −.24]	[.31, .42]	[.53, .62]	[.24, .36]	[.10, .23]	[−.24, −.12]	[.16, .28]	[.37, .47]	[.19, .31]	[.30, .42]	[−.50, −.39]	[.24, .36]				
20.A_Trust	3.25	0.76	−.02	−.00	.00	.06	.78 **	.09 **	−.31 **	.09 **	.06	.41 **	.04	−.18 **	.10 **	−.15 **	.42 **	.12 **	−.27 **	.03	.25 **			
			[−.09, .04]	[−.07, .06]	[−.06, .07]	[−.01, .12]	[.75, .80]	[.02, .15]	[−.36, −.25]	[.03, .16]	[−.00, .13]	[.36, .46]	[−.03, .10]	[−.24, −.12]	[.03, .16]	[−.21, −.08]	[.36, .47]	[.06, .18]	[−.33, −.21]	[−.03, .10]	[.19, .31]			
21.C_Responsibility	3.66	0.66	−.03	.03	.04	.25 **	.34 **	.80 **	−.36 **	.09 **	.09 **	.32 **	.48 **	−.21 **	.06	.27 **	.47 **	.62 **	−.35 **	.06	.27 **	.07 *		
			[−.09, .04]	[−.03, .10]	[−.03, .10]	[.19, .31]	[.29, .40]	[.78, .82]	[−.42, −.31]	[.03, .15]	[.02, .15]	[.26, .38]	[.43, .53]	[−.27, −.14]	[−.01, .12]	[.21, .33]	[.41, .52]	[.58, .66]	[−.41, −.29]	[−.01, .12]	[.21, .33]	[.00, .13]		
22.N_Emotional Volatility	2.78	0.86	−.03	−.05	−.02	−.03	−.37 **	−.28 **	.83 **	−.06	.05	−.14 **	−.12 **	.59 **	.00	−.05	−.45 **	−.29 **	.48 **	−.10 **	−.10 **	−.31 **	−.34 **	
			[−.10, .03]	[−.11, .02]	[−.09, .04]	[−.10, .03]	[−.42, −.31]	[−.34, −.22]	[.81, .85]	[−.13, .00]	[−.01, .12]	[−.21, −.08]	[−.18, −.06]	[.55, .63]	[−.06, .07]	[−.11, .02]	[−.50, −.40]	[−.35, −.23]	[.43, .53]	[−.17, −.04]	[−.17, −.04]	[−.37, −.25]	[−.39, −.28]	
23.O_Creative Imagination	3.66	0.80	.07 *	.06	.08 *	.34 **	.13 **	.06	−.11 **	.76 **	.19 **	.13 **	−.04	−.07 *	.42**	.30 **	.12 **	.12 **	−.14 **	.41 **	.37 **	.08 *	.10 **	−.06
			[.00, .13]	[−.01, .12]	[.02, .15]	[.28, .40]	[.07, .20]	[−.00, .13]	[−.17, −.04]	[.73, .78]	[.12, .25]	[.07, .19]	[−.10, .03]	[−.13, −.00]	[.37, .47]	[.24, .36]	[.05, .18]	[.06, .19]	[−.20, −.07]	[.36, .47]	[.31, .42]	[.01, .14]	[.04, .16]	[−.12, .01]

Note. Due to missing values, we used pairwise complete observations. Therefore, *N* varies between 909 and 907 for the correlations. RPT = Reverse Paraphrase Test (14 items), LPT = Literal Paraphrase Test (10 items); PMT = Proverb–Metaphor Test (14 items). *M* and *SD* are used to represent mean and standard deviation, respectively. Values in square brackets indicate the 95% confidence interval for each correlation. The confidence interval is a plausible range of population correlations that could have caused the sample correlation (Cumming 2014). * indicates *p* < .05. ** indicates *p* < .01. Correlations with RPT, LPT und PMT scores are based on sum scores. Correlations with the Big Five personality traits and facets scores are based on mean scores.

**Table 2:** CFAs and McDonald’s *ω* of the two tests for figurative language comprehension.

Measurement Model	RPT		LPT		
Sample	Sample 1	Sample 2	Sample 1	Sample 1 Adjusted	Sample 2
x^2^	105.386 **	100.604 **	400.008 ***	34.186	42.012
df	77	77	170	35	35
CFI	.910	.955	1	1	.998
RMSEA	.094	.065	.061	.000	.019
SRMR	.070	.054	.156	.048	.037
*ω*	.93	.93	1	.96	.97

Note. Sample 1: *n* = 363; Sample 2: *n* = 546. RPT = Reverse Paraphrase Test; LPT = Literal Paraphrase Test; *** *p* < .001, ** *p* < .05. Robust estimation was used for the RPT.

**Table 3:** CFAs and McDonald’s *ω* of the theoretical models for figurative language comprehension.

Theoretical Model	M_gfm_	M_cfm_	M_bfm_
x^2^	514.972 ***	364.154 ***	328.441 ***
df	252	251	238
CFI	.937	.973	.979
RMSEA	.041	.027	.025
SRMR	.040	.033	.030
AIC	11,389.644	11,164.323	11,135.733
BIC	11,620.637	11,400.128	11,434.098
*ω*	.92	.84 ^a^ .88 ^b^	.92 ^c^

Note. *N* = 909. *** *p* < .001. Robust estimation was used for CFA. ^a^: McDonald’s *ω* for RPT, ^b^: McDonald’s *ω* for LPT, ^c^: McDonald’s *ω* for the figurative comprehension factor.

**Table 4:** Comparison of different theoretical models for the allocation of figurative language comprehension.

Theoretical Model	FLC-g	FLC-gf	FLC-ak
x^2^	938.002 ***	942.560 ***	951.043 ***
df	575	575	575
CFI	.939	.938	.937
RMSEA	.040	.040	.040
SRMR	.049	.051	.051
AIC	20,785.322	20,790.157	20,798.558
BIC	21,159.264	21,164.098	21,172.500
*ω*	.89	.89	.89

Note. *n* = 450. *** *p* < .001. FCL = figurative language comprehension; FLC-g = figurative language comprehension—g factor; FLC-gf = figurative language comprehension—fluid intelligence; FLC-ak = figurative language comprehension—acquired knowledge, McDonald’s *ω* is only named for the figurative language comprehension factor.

## Data Availability

To make all analyses reproducible, we provide the analysis scripts and the codebook in an online repository: https://osf.io/e7msa/. Data are available from corresponding author on request.

## Supplementary Materials

The following supporting information can be downloaded at https://www.mdpi.com/article/10.3390/jintelligence12030029/s1. Table S1: Cohen’s Kappa Coefficients, Table S2: Descriptive Item Statistics, Table S3: Measurement Models RPT and LPT, Table S4: Theoretical Models Mgfm Mcfm Mbfm, Table S5: Theoretical Models FLC-g FLC-gf FLC-ak.
